# Gender equality in India hit by illiteracy, child marriages and violence: a hurdle for sustainable development

**DOI:** 10.11604/pamj.2017.28.178.13993

**Published:** 2017-10-26

**Authors:** Kishor Parashramji Brahmapurkar

**Affiliations:** 1 Department of Community Medicine, Government Medical College, Jagdalpur, Bastar, 494001 Chhattisgarh, India

**Keywords:** Gender equality, literacy, child marriage, violence, sex ratio, India

## Abstract

**Introduction:**

Gender equality is fundamental to accelerate sustainable development. It is necessary to conduct gender analyses to identify sex and gender-based differences in health risks. This study aimed to find the gender equality in terms of illiteracy, child marriages and spousal violence among women based on data from National Family Health Survey 2015-16 (NFHS-4).

**Methods:**

This was a descriptive analysis of secondary data of ever-married women onto reproductive age from 15 states and 3 UTs in India of the first phase of NFHS-4. Gender gap related to literacy and child marriage among urban and rural area was compared.

**Results:**

In rural area all states except Meghalaya and Sikkim had the significantly higher percentage of women's illiteracy as compared to male. Bihar and Madhya Pradesh had higher illiterate women, 53.7% and 48.6% as compared to male, 24.7% and 21.5% respectively (P < 0.000). Child marriages were found to be significantly higher in rural areas as compared to urban areas in four most populated states.

**Conclusion:**

There is a gender gap between illiteracy with women more affected in rural areas with higher prevalence of child marriages and poor utilization of maternal health services. Also, violence against women is showing an upward trend with declining sex-ratio at birth.

## Introduction

Gender refers to the socially constructed characteristics of women and men-such as the norms, roles and relationships that exist between them [1]. Gender inequality limits access to quality health services and contributes to avoidable morbidity and mortality rates in women, also gender inequality is unacceptable [1, 2]. The Convention on the Elimination of All Forms of Discrimination against Women requires that women are accorded rights equal to those of men (equality) and that women be able to enjoy all their rights in practice [3]. Realizing the significance of the issue, the policy makers have included the issue of gender equality as one among seventeen Sustainable Development Goals (Goal-5) [3]. Encouraging gender equality is fundamental to accelerating sustainable development [3]. Some of the elements of gender equality are to abolish all forms of violence against all women and girls along with all destructive practices, such as child marriage and guarantee worldwide access to reproductive health and reproductive rights [3]. Social determinants of health such as education and gender equality are significantly responsible for health-seeking behavior and overall health outcomes. It has been known that improved education, partly reproduced by higher literacy rates is associated with higher incomes and better health indicators such as lower infant mortality rates (IMRs) and lesser population growth rate. Education of families, particularly of women has a 'multiplier effect' on development [4]. Child marriage and adolescent pregnancy, gender-based violence are among the many barriers that stand in the way of woman's' fully exercising their right to education [4]. No education limits hopes, declines family income, diminishes health, puts women at risk of trafficked and exploitation and bounds the economic advancement of entire countries [5]. Education for girls and women is the single most successful way to progress the lives of individual families as well as to bring economic expansion to poor communities worldwide [5]. Globally 31 million girls are out of school and two-thirds of illiterate adults are women [6]. Poverty, adolescent pregnancy, child marriage and prejudiced gender norms are some of the reasons that prevent girls from going to school [6]. Globally 39000 child marriages (marriage before the age of 18) occur daily and it is more common to young girls [7]. Child marriages not only contribute to illiteracy but also have complications related to pregnancy and childbirth. These complications are the leading cause of death in young women aged 15-19 [7]. Child marriages also make girls more susceptible to intimate partner violence(IPV) [7]. Marrying girls less than 18 years old has been embedded in gender discrimination, cheering premature and uninterrupted child-bearing and giving the predilection for boys' education [8]. Current worldwide prevalence figures indicate that about 1 in 3 (35%) of women worldwide have experienced intimate partner violence (IPV) [9]. Gender inequality and poor education are some of the reasons for IPV [9]. IPV can lead to unplanned pregnancies, induced abortions, gynecological problems and sexually transmitted infections [9]. It is necessary to disaggregate data and conduct gender analyses to identify sex and gender-based differences in health risks [1]. The overall aim of the study was to find the gender equality in terms of illiteracy, child marriages and spousal violence among women based on data from National Family Health Survey 2015-16 (NFHS-4).

## Methods

**Study design**: The present study was a cross-sectional secondary data analysis of information that has been available from the first phase of National Family Health Survey 2015-16 (NFHS-4) [10]. NFHS-4, has given information on population, health and nutrition each State/Union Territory.

**Setting**: Primary data onto NFHS-4 had been collected from January 2015 to December 2015. The Ministry of Health and Family Welfare, Government of India assigned International Institute for Population Sciences, Mumbai as the nodal organization to conduct NFHS-4. In the first phase of NFHS-4, 15 states and 3 union Territories were covered (56% of total population). Fifteen States/Union Territories had been selected for study purpose was those, which were covered in first phase of NFHS-4; Maharashtra (MH), Bihar (BR), West Bengal (WB), Madhya Pradesh (MP), Tamil Nadu (TN), Karnataka (KA), Andhra Pradesh (AP), Telangana (TS), Assam (AS), Haryana (HR), Uttarakhand (UK), Tripura (TR), Meghalaya (ML), Manipur (MN) and Sikkim (SK). UTs were Goa (GA), Puducherry (PY) and Andaman and Nicobar (AN) [11].

**Sample size**: First phased of NFHS-4 had collected information about 2,91,431 households, 3,37,658 women and 48,342 men.

**Variables**: NFHS-4 had provided updates and evidence of developments in key population, health and nutrition indicators out of which literacy, child marriage; adolescent reproductive health, maternal health, domestic violence and sex-ratio had been included for the study purpose.

**Data analysis**: First the data for the above-mentioned variables of all States/UTs has been entered in Microsoft Office Excel worksheet and the gender gap for illiteracy was calculated using Chi-square test for urban and rural area. Then the prevalence of child marriages among girls was compared for area of residence (Urban versus Rural) and Chi-square value was calculated using Epi Info. For statistical tests, P < 0.05 was taken as the significant level. Data were then presented using tables, bar and line diagrams.

**Ethical considerations**: The study had utilized freely-available record available on the website of the following organization: the National Family Health Survey (NFHS)-4 [10], National Crime Record Bureau (NCRB) [12] and Census 2011 [13]. Because publicly-available database was used in this analysis, no ethical approval was sought.

**Definitions**: Literate: a person that can read and write with understanding in any language [13]. Child marriage: according to the prohibition of child Marriage Act, 2006 a child is a male who has not completed twenty-one years of age and a female who has not completed eighteen years of age. Child marriage is a contract between any two people of which either one or both party are a child [14]. Full antenatal care was defined as at least four antenatal visits, at least one tetanus toxoid (TT) injection and iron-folic acid tablets or syrup took for 100 or more days [10] . Intimate partner violence refers to behavior by an intimate partner or ex-partner that causes physical, sexual or psychological harm, including physical aggression, sexual coercion, psychological abuse and controlling behaviors [9].

## Results

Total population covered by phase I of NFHS-4 was 678.2 million over 15 states and 3 Union Territories (UTs).

**Women's illiteracy (%) as compared to men's illiteracy (%) in urban and rural areas**: Women's illiteracy in an urban area has been found to be significantly higher in 8 states and 2 UTs as compared to men's illiteracy and in the rural area; it was significantly higher in 13 states and 1 UT respectively (Table 1). In an urban area, Bihar had a higher percentage of illiterate women (29.5%) as compared to men (11.2%), (P < 0.001), followed by Andhra Pradesh and Madhya Pradesh (MP). In the rural area, all states except Meghalaya and Sikkim had a significantly higher percentage of women's illiteracy as compared to male. Bihar and MP had higher illiterate women, 53.7% and 48.6% as compared to male, 24.7% and 21.5% respectively. (P < 0.000). Bihar and MP state with the population of total 176.7 million had higher women illiteracy in both urban and rural areas. This indicates gender inequality in education.

**Table 1:** Distribution of states of India according to the gender gap in illiteracy among urban area as compared to rural area

Sr. No.	State	Population in million	Women's illiteracy (%)	Men's illiteracy (%)	χ2	*P*value	Women's illiteracy (%)	Men's illiteracy (%)	χ2	*P* value
			Urban	Urban			Rural	Rural		
1	Maharashtra	112.4	14.1	5.6	3.167	0.037	25.2	8.8	8.404	0.002
2	Bihar	104.1	29.4	11.2	9.143	0.001	53.7	24.7	16.45	0
3	West Bengal	91.3	20.6	16.1	0.409	0.261	33.1	20.3	3.557	0.03
4	Madhya Pradesh	72.6	22.5	11.3	3.704	0.027	48.6	21.5	14.96	0
5	Tamil Nadu	72.1	14.4	8.3	1.293	0.128	27.1	13.8	4.65	0.016
6	Karnataka	61.1	18.2	10	2.14	0.072	36.2	18.8	6.745	0.005
7	Andhra Pradesh	49.6	25.1	9.8	7.098	0.004	42.6	26.4	5.112	0.012
8	Telangana	35.1	20.7	9.2	4.335	0.019	47.6	23.5	11.64	0
9	Assam	31.2	13	6.8	1.516	0.109	30.8	19.3	2.936	0.043
10	Haryana	25.4	19.7	7	5.917	0.007	27.9	11.1	7.952	0.002
11	Uttarakhand	10.1	18.3	7.6	4.173	0.021	26.4	10.4	7.493	0.003
12	Tripura	3.7	11.6	4.8	2.234	0.068	23	13	2.744	0.049
13	Meghalaya	3	6.6	4.3	0.164	0.343	20.4	19.2	0.001	0.486
14	Manipur	2.9	10.1	2.6	3.552	0.03	18.3	4.8	7.647	0.003
15	Goa	1.5	12	6.5	1.206	0.137	9.2	3.4	1.952	0.081
16	Puducherry	1.2	14.9	10.2	0.554	0.228	15.2	3.9	6.141	0.007
17	Sikkim	0.6	10.5	6.7	0.499	0.24	14.8	10	0.665	0.208
18	Andaman and Nicobar	0.4	11	3.9	2.698	0.05	19.8	16.7	0.148	0.35

**Child marriages to women (%) in urban and rural areas**: Child marriages to women were found to be significantly higher in rural areas as compared to urban areas in 4 most populated states (Maharashtra, Bihar, West Bengal and MP). West Bengal had a higher percentage of child marriages in both urban and rural area, 27.7% and 46.3% and the difference between urban and rural area was significant (P < 0.005) followed by Bihar, 26.9% and 40.9% of child marriages among urban and rural women respectively. (P < 0.026), though no statistically significant difference was observed in Andhra Pradesh, Assam and Tripura, the prevalence of child marriage among women was more than 20% in urban and more than 33% in rural areas of concerned states. The above findings point of the harmful practice of child marriages to women prevalent in most of the states and UTs of India. Goa was the only exception with child marriages significantly higher in an urban area as compared to a rural area as shown in Table 2.

**Table 2:** Child marriages of girls in rural and urban areas of some states and UTs of India

Sr. No.	State	Child marriages in women (%)		χ2	*P* value
		Urban	Rural		
1	Maharashtra	18.8	31.5	3.636	0.028
2	Bihar	26.9	40.9	3.771	0.026
3	West Bengal	27.7	46.3	6.644	0.005
4	Madhya Pradesh	16.6	35.8	8.566	0.002
5	Tamil Nadu	13	18.3	0.7	0.201
6	Karnataka	17.9	27	1.884	0.085
7	Andhra Pradesh	26.3	35.5	1.575	0.105
8	Telangana	15.7	35	8.848	0.001
9	Assam	23.9	33.9	1.971	0.08
10	Haryana	19.6	17.8	0.021	0.442
11	Uttarakhand	12.2	14.8	0.11	0.37
12	Tripura	25.6	34.8	1.595	0.104
13	Meghalaya	7.8	19.3	4.706	0.015
14	Manipur	11	14.3	0.239	0.312
15	Goa	14.8	2.7	7.716	0.003
16	Puducherry	10.9	10.2	0.005	0.473
17	Sikkim	16.1	13.6	0.089	0.382
18	Andaman and Nicobar	11.9	20.4	2.077	0.075

**Reproductive health**: Full Antenatal care (ANC) was lower in a rural area of states, Bihar (03%), followed by Tripura (6.8%) and MP (8.3%) as compared to the rural area of Tamil Nadu (43.8%). Also, full ANC coverage was found to be lower in an urban area of Bihar (6.6%), Tripura (9.8%), Uttarakhand (15.6%) and MP (19.5%) as compared to Telangana and Tamil Nadu with full ANC coverage of 47.7% and 46.3% respectively. Postnatal care (PNC) to mothers within 2 days of delivery was found lower in rural area of states with Bihar state in which only 41.1% of mothers received PNC from health personnel followed by Meghalaya (42.6%), Uttarakhand (49.1%) and MP (50.3%) as compared to better Post Natal Care in state of Telangana (79.1%) and Andhra Pradesh (77.8%) among states and 90.5% among UT (Goa).

**Figure 1:**
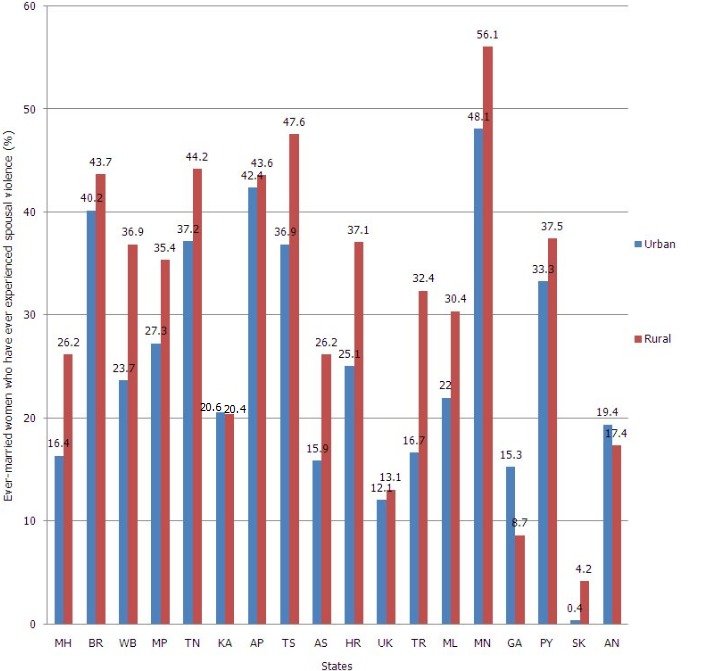
Distribution of states and UTs of India according to percentage of ever-married women who have experienced spousal violence

**Figure 2:**
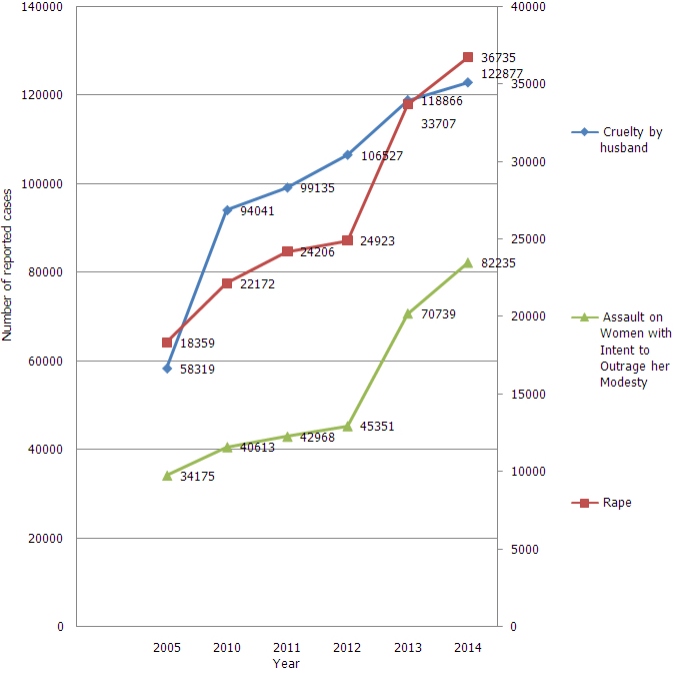
Distribution of number of cases reported as per National Crime Record Bureau (NCRB), India, under head of cruelty by husband and rape from the year 2005 to 2014

**Ever-married women that have ever experienced spousal violence (%) and adolescent pregnancy**: The overall percentage of spousal violence among ever-married women was 25.2% in urban area and 31.2% in rural area. Spousal violence was higher in the rural area of Manipur (56.1%) followed by Telangana (47.6%), Tamil Nadu (44.2%) and Bihar (43.7%). Similar observations were noted in an urban area as shown in Figure 1. Figure 2 shows a number of cases reported under the head of cruelty by husband and rape. There was an upward trend in a number of cases reported to the head of cruelty by husband and rape from the year 2010 to 2014. Percentage of adolescent pregnancy was higher in Tripura and West Bengal in the rural area (20.7% and 20.6%) as compared to an urban area (13.7% and 12.4%) respectively.

**Figure 3:**
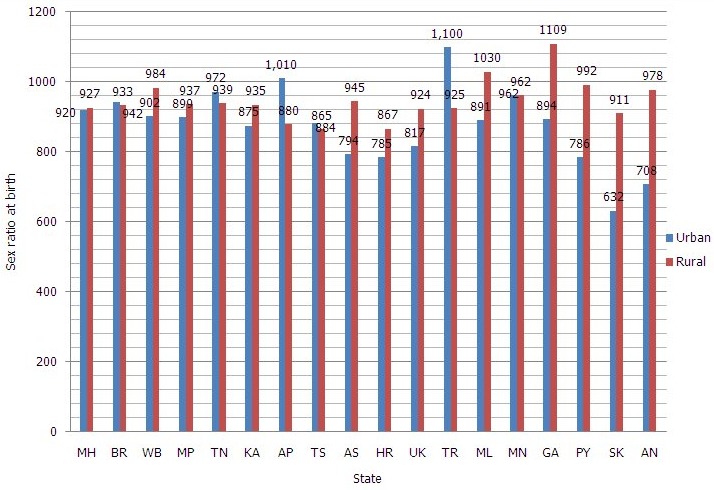
Distribution of states and UTs of India according to sex-ratio at birth in urban and rural areas

**Sex ratio at birth for children born in the last five years (females per 1,000 males)**: Sex ratio at birth was better in the rural area except for Telangana (865 females/1000 males), Haryana (867 females/1000 males) and Andhra Pradesh (880 females/1000 males). In urban areas, it was lower than 900 females/1000 males in all UTs and 8 states. It was lower in Sikkim (632 females/1000 males), Andaman and Nicobar (708 females/1000 males) and Haryana (785 females/1000 males) as summarized in Figure 3.

## Discussion

In present study female literacy has been significantly lower as compared to male literacy in rural areas of 15 states and 3 UTs except for Meghalaya and Sikkim state and Goa and Andaman and Nicobar UTs. The reason for this may be several parents did not have permitted their female children to go to schools and another reason could be child marriage of girls [15]. Another reason might be that most people are below the poverty line and weren't conscious that children should get the free education according to the law [15]. Hence there is a gender gap between educations. The 11^th^ five-year plan had decided to reduce the gender gap in literacy to 10% points by 2012 [16]. However in present study gender gap was observed in rural areas of all states except Meghalaya and Sikkim. Also according to censuses held in 2001 and 2011, the percentage of female literacy in the country was 54.16% and 65.46% respectively. An increase in 11.3% during the period 2001-2011, however, this was 3.6% lower than that during the period of 1991-2001. This declining trend over a decade is a matter of concern for sustainable development as it affects women empowerment [17]. In a country like India, literacy is the core basis of social and economic growth. Though the government has made an act that each child under the age of 14 should get free education, the setback of illiteracy is still at large [15]. Similarly, Lailulo YA et al had observed gender gap between education in the Ethiopia and also noted that educated women with educational attainment of primary education and above are less likely got married at an early age than those who are uneducated [18]. Raj Anita et al had studied the prevalence of child marriage using National Family Health Survey-3 data and had found that the maximum frequency of child marriage among women having less than a secondary education and residing in the rural area [19]. Similar findings were noted in NFHS-4 data phase 1, among top 4 most populated states. West Bengal and Bihar had a higher percentage of child marriage, 46.3 and 40.9% respectively in rural areas. David R et al had also similar observations related to higher prevalence of child marriage to girls with less education and residing in rural areas [20]. Adolescent pregnancy or 'motherhood in childhood' is one of the gravest health hazards to young women in India. Patra S et al had observed that stillbirth and abortion were more widespread among younger adolescents and the proportion of live births (vs. stillbirth or abortion) was also advanced among women having 10 years or more education [21]. As per UNFPA, the girl with adolescent pregnancy bears end of her education along with shrinking away from her job prospects and her vulnerability to poverty and exclusion increases [22]. Impediments from pregnancy and childbirth were the leading cause of death among adolescent girls [22]. In the present study, it was observed that coverage of full antenatal care (ANC) in states was the lowest in rural Bihar 03% compared to rural Tamil Nadu, 43.8%.Ahmed S et al had observed that women with complete primary education are almost three times more likely to have made at least four ANC visits [23]. Bihar had 53.7% of women illiteracy in the rural area as compared to Tamil Nadu, 27.1%. Kawaguchi L et al. had noted that women that married young were less likely to utilize ANC [24]. Bihar had 40.9% of child marriages to the rural area as compared to Tamil Nadu, 18.3%. Birmeta K et al had observed that women with education were more than twice likely to attend ANC as compared with those who had no education [25]. Ensuring universal accesses to sexual and reproductive health and reproductive rights as agreed in accordance with the Programme of Action of the International Conference on Population and Development and the Beijing Platform for Action and the outcome documents of their review conferences. 'Gender equality means that the different behaviors, aspirations, and needs of women and men are considered, valued and favored equally' [26]. Gender equality is a matter of human rights. It is also a driver of development progress. Gender equality, rooted in human rights, is increasingly recognized both as an essential development goal on its own and as vital to accelerating sustainable development overall [27]. Sex ratio is defined as the number of females per 1000 males in the population and is an important social indicator to measure the extent of prevailing equity between males and females [28]. Though the overall sex ratio of the Country is showing a trend of improvement, the child sex ratio is showing a declining trend, which is a matter of concern. Child sex ratio (0-6 years) at country level was 945 in 1991, 927 in 2001 and has now declined to 914 in Census 2011 [27]. The sex ratio of birth is an indicator of the discrimination against the girl child and dreadful crimes such as female feticide. As per NFHS-4 child sex ratio was the lowest in urban Sikkim, 632 followed by Andaman and Nicobar and Haryana with child sex-ratio of 708 and 785 respectively. Gender inequality manifests itself in various forms, the most obvious being the tendency towards the continuously declining female ratio of the population of the last few decades [29]. Further research is needed to study the factors associated with declining female ratio of the population.

**Limitations**: This study had not covered all states and UTs of India. The datasets were not available for NFHS-4 at present, so detailed analysis was not done.

## Conclusion

There is the gender gap between illiteracy with women more affected in rural areas with higher prevalence of child marriages and poor utilization of maternal health services. Also, the violence against women is showing an upward trend with declining sex-ratio at birth.

### What is known about this topic

- 11^th^ five year plan of India had decided to reduce the gender gap between literacy to 10% points by 2012;
- Child sex ratio of India was 919 females/1000 males (as per Census 2011).

### What this study adds

- 11 out of 15 States/Union Territories (73%) had gender gap of more than 10% in literacy ranging from 10% to 29% in the rural area as compared to urban areas in 6 States/Union Territories (40%) with the range of 10.7% to 18.2%;
- But as per the analyses of primary data of NFHS-4 which was collected from January 2015 to December 2015, child sex ratio of India is declining with lowest, 632/1000 females in urban area of Sikkim and below 800/1000 females in urban area of 5 states States/Union Territories.

## Competing interests

The author declares no competing interests.

## References

[1] WHO (2015). Gender. WHO.

[2] (2014). United Nation Women’s Rights are Human Rights.

[3] United Nations Development Programme (2017). GOAL 5 TARGETS.

[4] United Nations Educational, Scientific and Cultural Organization (UNESCO) (2017). Building peace in the minds of men and women.

[5] World Education Girls' and Women's Education.

[6] Right to Education Initiative (2017). Girls & women.

[7] WHO (2017). Child marriages: 39 000 every day.

[8] UNICEF (2016). Child marriage.

[9] WHO (2016). Violence against women.

[10] International Institute for Population Sciences (2015).

[11] List of State Abbreviation

[12] (2015). TABLE 1.3 Cases Reported & Rate of Cognizable Crimes (IPC) under Different Crime Heads During 2015 and Decadal & Quinquennial Percentage Changes.

[13] 07Literacy (2011). STATUS OF LITERACY.

[14] Childline India Foundation (2006). CHILD Protection & Child Rights » IV. National Mechanisms » Child Related Legislations » Prohibition of Child Marriage Act, 2006.

[15] (2011). Census 2011 Literacy in India.

[16] (2011). Final_PPT_2011_chapter6.pdf. State of literacy.

[17] Frame1 (2001). Female Literacy in India.

[18] Lailulo YA, Sathiya Susuman A, Blignaut R (2015). Correlates of gender characteristics, health and empowerment of women in Ethiopia. BMC Womens Health..

[19] Raj A, Saggurti N, Balaiah D, Silverman JG (2009). Prevalence of child marriage and its effect on fertility and fertility-control outcomes of young women in India: a cross-sectional, observational study. The Lancet..

[20] Hotchkiss DR, Godha D, Gage AJ, Cappa C (2016). Risk factors associated with the practice of child marriage among Roma girls in Serbia. BMC Int Health Hum Rights..

[21] Shraboni Patra (2016). Motherhood in childhood: addressing reproductive health hazards among adolescent married women in India. Reprod Health..

[22] UNFPA - United Nations Population Fund Adolescent pregnancy.

[23] Ahmed S, Creanga AA, Gillespie DG, Tsui AO (2010). Economic Status, Education and Empowerment: Implications for Maternal Health Service Utilization in Developing Countries. PLoS One..

[24] Kawaguchi L, Fouad NAM, Chiang C, Elshair IHH, Abdou NM, El Banna SR (2014). Dimensions of women's empowerment and their influence on the utilization of maternal health services in an Egyptian village: a multivariate analysis. Nagoya J Med Sci..

[25] Birmeta K, Dibaba Y, Woldeyohannes D (2013). Determinants of maternal health care utilization in Holeta town, central Ethiopia. BMC Health Serv Res..

[26] United Nation (2011). women-s-empowerment-principles.

[27] UNDP (2014). Gender Equality Strategy 2014-2017.

[28] 06Gender Composition Gender Composition.

[29] National policy for the empowerment of wmomen (2011). National Policy For The Empowerment Of Women, 2001.

